# High preoperative white blood cell count determines poor prognosis and is associated with an immunosuppressive microenvironment in colorectal cancer

**DOI:** 10.3389/fonc.2022.943423

**Published:** 2022-07-29

**Authors:** Meilin Weng, Wenling Zhao, Ying Yue, Miaomiao Guo, Ke Nan, Qingwu Liao, Minli Sun, Di Zhou, Changhong Miao

**Affiliations:** ^1^ Department of Anesthesiology, Zhongshan Hospital, Fudan University, Shanghai, China; ^2^ Department of Anesthesiology, Shanghai Cancer Center, Fudan University, Shanghai, China; ^3^ Shanghai Key Laboratory of Perioperative Stress and Protection, Fudan University, Shanghai, China

**Keywords:** high preoperative WBC, preoperative leukocytosis, long-term prognosis, colorectal cancer, propensity score matching, tumor microenvironment

## Abstract

**Background:**

The correlation between high white blood cell (WBC) count and poor prognosis has been identified in various types of cancer; however, the clinical significance and immune context of WBC count in colorectal cancer remains unclear.

**Methods:**

Between February 2009 and November 2014, 7,433 patients at the Shanghai Cancer Center who had undergone elective surgery for colorectal cancer were enrolled in this retrospective cohort study. Patients were divided into two groups: low and high preoperative WBC groups. Propensity score matching was used to address the differences in baseline characteristics. The Kaplan–Meier method and Cox regression analysis were used to identify independent prognostic factors in colorectal cancer patients. Tumor-infiltrating immune cells in the high and low preoperative WBC groups were compared using immunohistochemical staining.

**Results:**

Of the 7,433 patients who underwent colorectal cancer surgery and were available for analysis, 5,750 were included in the low preoperative WBC group, and 1,683 were included in the high preoperative WBC group. After propensity score matching, 1,553 patients were included in each group. Kaplan–Meier survival curves showed that a high preoperative WBC count was associated with a decreased overall survival (P = 0.002) and disease-free survival (P = 0.003), and that preoperative WBC count was an independent risk factor for overall survival (hazard ratio, 1.234; 95% confidence interval, 1.068–1.426; P = 0.004) and disease-free survival (hazard ratio, 1.210; 95% confidence interval, 1.047–1.397, P = 0.01). Compared to the low preoperative WBC group, the high preoperative WBC group exhibited higher expression of regulatory T cells (P = 0.0034), CD68^+^ macrophages (P = 0.0071), and CD66b^+^ neutrophils (P = 0.0041); increased expression of programmed cell death protein 1 (P = 0.005) and programmed cell death ligand 1 (P = 0.0019); and lower expression of CD8^+^ T cells (P = 0.0057) in colorectal cancer patients.

**Conclusions:**

Our research indicates that a high preoperative WBC count is a prognostic indicator in colorectal cancer patients and is associated with an immunosuppressive tumor microenvironment, which could aid in future risk stratification.

## Introduction

Colorectal cancer (CRC) is the third most common tumor in the world and one of the leading causes of cancer-related deaths worldwide (1). In China, CRC ranks fifth among the main causes of death caused by cancer among men and women; however, the death rates from CRC have been on the rise in recent decades (2, 3). Although great progress has been made in surgery, chemotherapy, and radiotherapy, the mortality rate of CRC remains high. A large proportion of CRC patients still develop resistance to chemotherapy and eventually relapse within two years after undergoing surgery (4). Thus, a prognostic indicator is urgently needed to predict the outcome of CRC patients, which is very important for risk stratification and determining treatment strategies (5).

CRC is an inflammation-related tumor characterized by the infiltration of heterogeneous immune cells into tumor microenvironment and peripheral hematological disorders (6), which create a complex microenvironment that allows for the development of tumors (7). During acute inflammation, white blood cells (WBCs) are considered the first line of defense against microbial infection, protecting the host from pathogens (8). However, in the state of long-term activation, continuous production of growth factors and reactive oxygen species may lead to permanent genomic changes and hinder the recruitment of lymphocytes by interacting with the DNA of the proliferating epithelium. Moreover, leukocytosis inhibits the activation of CD8^+^ tumor infiltrating lymphocytes by upregulating programmed death protein 1 (PD-1) on T lymphocytes and myeloid cells (7, 9). In malignant diseases, leukocytes and neutrophils can dilate locally and throughout the body, promoting anti-cancer treatment resistance and tumor progression through angiogenesis, invasion, and inhibitory factors (10). Tumor-infiltrating immune cells and inflammatory cells are the main components of the tumor microenvironment (TME) and are critical for the immune function of the host and the biological behavior of the tumor (11). Chronic inflammation is thought to lead to the development of various malignant lesions and an increased risk of recurrence (12).

As indicators of prognosis, various serum molecular markers, such as basophil, neutrophil, and lymphocyte counts, are easy to acquire from conventional preoperative examinations and are useful in diagnosing and evaluating treatment and predicting the prognosis of CRC patients (13–15). The correlation between a high WBC count and poor prognosis has been identified in various cancer types, such as oropharyngeal, cervical, and esophageal cancer (16–18). Although several groups have reported the adverse effects of peripheral leukocytosis on the prognosis of patients with various malignant tumors, this is limited by a retrospective mismatch, uneven treatment options, short follow-up, lack of multivariable analysis, and a small number of patients (5, 19, 20). The mechanism underlying this phenomenon has not yet been clarified.

Therefore, the purpose of this study was to investigate the prognostic value of preoperative WBC count on overall survival (OS) and disease-free survival (DFS) after CRC surgery in a larger matched sample cohort and the functional relevance of WBC in an immunological context. We speculated that high preoperative WBC count predicts a poorer survival outcome in CRC patients undergoing selective surgery and is associated with an immunosuppressive TME in CRC.

## Materials and methods

### Study design

This retrospective study was approved by the Ethics Committee of the Shanghai Cancer Center at Fudan University in Shanghai, China (IRB2105235-6). All the participants signed an informed consent form.

### Study population

**Figure 1:**
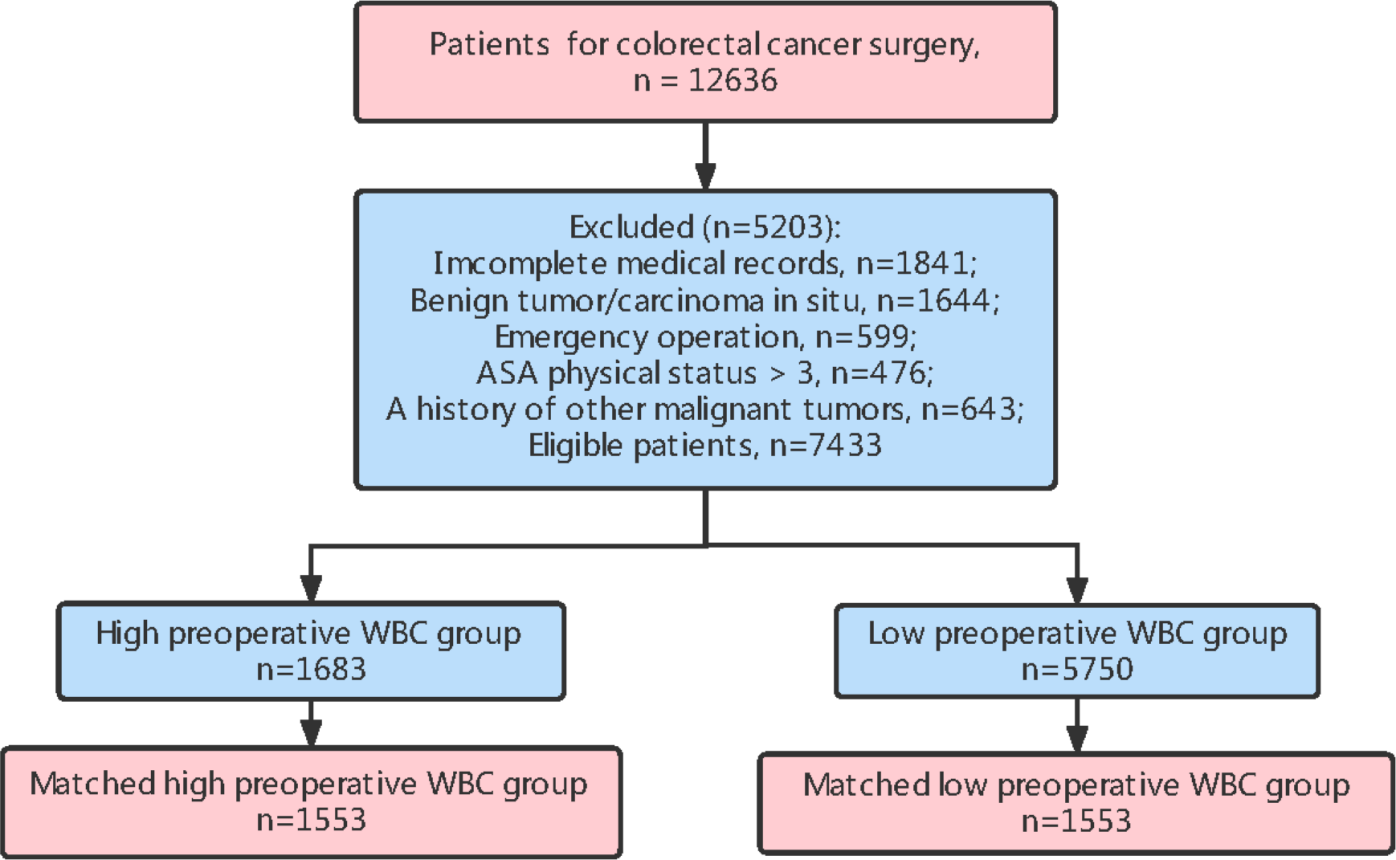
A. Flow chart of patient selection.

Between February 2009 and November 2014, 12,636 patients underwent elective surgery for CRC, and 7,433 patients with clinical characteristic data, OS records, and DFS records were included in this study (**Figure 1**). The inclusion criteria were as follows CRC diagnosed according to histological evidence, patients undergoing elective radical surgery for CRC, and patients older than 20 years.

We excluded patients with incomplete medical information, benign tumors or carcinoma *in situ*, emergency surgery, an American Society of Anesthesiologists physical status of > 3, or those with a history of other malignant tumors during the initial visit (**Figure 1**). According to the preoperative WBC count, patients were classified into a high preoperative WBC group (WBC count ≥ 7,000/µL) and low preoperative WBC group (WBC count < 7,000/µL). The cut-off value for WBC count was calculated using the receiver operating characteristic (ROC) curve; the threshold was associated with an increased risk of postoperative mortality and was within the normal range of the WBC count (21).

### Variables and outcomes

We reviewed and recorded the following variables from the clinical information system of the Shanghai Cancer Center: sex, age, preoperative adjuvant chemotherapy, surgical approach, tumor location, tumor histology, vascular tumor thrombus, nerve invasion, surgical margin positivity, T, N, M, and TNM stages, infiltrating lymph nodes > 12, number of cancer nodules ≥1, preoperative hemoglobin, surgery again within 30 days, death, intraoperative transfusion, and blood loss.

The main endpoints of the study were OS and DFS. OS was defined as the interval between the date of diagnosis and the date of death from any cause. DFS was defined as the interval between the date of diagnosis and the date of recurrence, metastasis, occurrence of a secondary primary tumor, or death.

### Immunohistochemical staining

Among patients underwent elective surgery for CRC in 2014, 30 patients were randomly selected from the high preoperative WBC group and 30 patients from the low preoperative WBC group. These 60 CRC tissues were used for IHC. Paraffin-embedded tissues were stained with antibodies. Two experienced pathologists determined the staining score. Six high-power fields (×200 magnification) were randomly counted by two independent pathologists (each experts in three fields), and the densities of CD8^+^ T cells, Foxp3^+^ regulatory T (Treg), CD68^+^ macrophages, CD66b^+^ neutrophils, PD-1^+^ cells, and programmed cell death ligand 1 (PD-L1)^+^ cells were recorded. The immunohistochemical antibodies used are listed in **Supplementary Table 1**.

### Statistical analysis

In this study, the baseline characteristics of the patients were expressed by the values and percentages of the classified variables. The chi-square test was used to evaluate differences in baseline characteristics between the two groups. To reduce the possible confounding effect of each variable and the difference in baseline characteristics between the two groups, propensity score matching was performed. The paired variables were sex, age, preoperative adjuvant chemotherapy, tumor histology, TNM stage, and infiltrating lymph nodes > 12. The R software package “MatchIt” was used to match the propensity score. After matching, 1,553 patients were included in each group (**Figure 1**; **Table 1**).

**Table 1:** Patients baseline Characteristics in the total study cohort and the propensity score matched cohort.

Variables	Overall patients		P value	Matched patients		P value
	Low pre-WBC(n=5750)	High pre-WBC(n=1683)		Low pre-WBC(n=1553)	High pre-WBC(n=1553)	
**Sex, n (%)**			<0.001			0.472
Male	3315 (57.7)	1094 (65.0)		1031 (66.4)	1012 (65.2)	
Female	2435 (42.3)	589 (35.0)		522 (33.6)	541 (34.8)	
**Age, n (%)**			0.446			0.917
≤44	799 (12.8)	269 (14.3)		174 (11.2)	175 (11.3)	
45-54	1247 (20.0)	386 (20.5)		338 (21.8)	322 (20.7)	
55-64	2263 (36.3)	652 (34.6)		569 (36.6)	563 (36.3)	
65-74	1342 (21.5)	404 (21.4)		335 (21.6)	346 (22.3)	
>75	585 (9.4)	174 (9.2)		137 (8.8)	147 (9.5)	
**Pre-chemotherapy, n (%)**			<0.001			0.673
No	5239 (91.1)	1587 (94.3)		1477 (95.1)	1482 (95.4)	
Yes	511 (8.9)	96 (5.7)		76 (4.9)	71 (4.6)	
**Surgical approach, n (%)**			0.805			0.546
Laparotomy	5296 (92.1)	1547 (91.9)		1437 (92.5)	1428 (92.0)	
Laparoscopy	454 (7.9)	136 (8.1)		116 (7.5)	125 (8.0)	
**Tumor location, n (%)**			<0.001			0.070
Rectum	3381 (54.2)	909 (48.2)		820 (52.8)	754 (48.6)	
Left-side colon	1283 (20.6)	433 (23.0)		326 (21.0)	353 (22.7)	
Right-side colon	1483 (23.8)	509 (27.0)		393 (25.3)	422 (27.2)	
Entire colon	12 (0.2)	4 (0.2)		0 (0.0)	2 (0.1)	
Transverse colon (can’t tell right or left)	77 (1.2)	30 (1.6)		14 (0.9)	22 (1.4)	
**Tumor histology, n (%)**			<0.001			0.424
Adenocarcinoma	5034 (87.5)	1410 (83.8)		1331 (85.7)	1340 (86.3)	
Mucinous adenocarcinoma	641 (11.1)	240 (14.3)		199 (12.8)	198 (12.7)	
Signet-ring cell carcinima	75 (1.3)	33 (2.0)		23 (1.5)	15 (1.0)	
**Vascular tumor thrombus, n (%)**			0.080			0.276
Negative	4549 (79.1)	1298 (77.1)		1229 (79.1)	1204 (77.5)	
Positive	1201 (20.9)	385 (22.9)		324 (20.9)	349 (22.5)	
**Nerve invasion, n (%)**			0.691			0.707
Negative	4722 (82.1)	1375 (81.7)		1282 (82.5)	1274 (82.0)	
Positive	1028 (17.9)	308 (18.3)		271 (17.5)	279 (18.0)	
**Surgical Margin positivity, n (%)**			0.035			0.038
No	5677 (98.7)	1650 (98.0)		1537 (99.0)	1523 (98.1)	
Yes	73 (1.3)	33 (2.0)		16 (1.0)	30 (1.9)	
**T stage, n (%)**			<0.001			0.748
T1	486 (8.4)	98 (5.8)		105 (6.7)	91 (5.8)	
T2	1121 (19.5)	254 (15.1)		257 (16.5)	238 (15.3)	
T3	246 (4.3)	86 (5.1)		73 (4.7)	75 (4.8)	
T4	3657 (63.6)	1201 (71.4)		1080 (69.5)	1115 (71.8)	
Tx	240 (4.2)	44 (2.6)		38 (2.4)	34 (2.2)	
**N stage, n (%)**			0.421			0.431
N0	3204 (55.7)	912 (54.2)		864 (55.6)	848 (54.6)	
N1	1643 (28.6)	487 (28.9)		422 (27.2)	453 (29.3)	
N2	903 (15.7)	284 (16.9)		267 (17.2)	252 (16.2)	
**M stage, n (%)**			0.002			0.389
M0	5465 (95.0)	1567 (93.1)		1472 (94.8)	1461 (94.1)	
M1	285 (5.0)	116 (6.9)		81 (5.2)	92 (5.9)	
**TNM stage, n (%)**			<0.001			0.923
I	1183 (20.6)	259 (15.4)		244 (15.7)	242 (15.6)	
II	1661 (28.9)	576 (34.2)		555 (35.7)	545 (35.1)	
III	2439 (42.4)	705 (41.9)		653 (42.0)	656 (42.2)	
IV	285 (5.0)	116 (6.9)		81 (5.2)	92 (5.9)	
Unknown	182 (3.2)	27 (1.6)		20 (1.3)	18 (1.2)	
**Infiltrating lymph nodes > 12, n (%)**			<0.001			0.176
No	1303 (22.7)	310 (18.4)		294 (18.9)	265 (17.1)	
Yes	4447 (77.3)	1373 (81.6)		1259 (81.1)	1288 (82.9)	
**Number of cancer nodules ≥ 1, n (%)**			0.600			0.836
No	4969 (86.4)	1446 (85.9)		1334 (85.9)	1338 (86.2)	
Yes	781 (13.6)	237 (14.1)		219 (14.1)	215 (13.8)	
**Preoperative Hemoglobin, g/L**			<0.001			0.005
<90	331 (5.8%)	150 (8.9%)		93 (6.0%)	134 (8.6%)	
≥90	5419 (94.2%)	1533 (91.1%)		1460 (94.0%)	1419 (91.4%)	
**Results**						
**Surgery again within 30 days, n (%)**			0.468			0.289
No	5646 (98.2)	1657 (98.5)		1520 (97.9)	1528 (98.4)	
Yes	104 (1.8)	26 (1.5)		33 (2.1)	25 (1.6)	
**Death, n (%)**			0.003			0.007
No	4391 (76.4)	1225 (72.8)		1208 (77.8)	1144 (73.7)	
Yes	1359 (23.6)	458 (27.2)		345 (22.2)	409 (26.3)	
**Blood Transfusion, n (%)**			<0.001			0.001
No	5635 (98.0)	1618 (96.1)		1525 (98.2)	1493 (96.1)	
Yes	115 (2.0)	65 (3.9)		28 (1.8)	60 (3.9)	
**Blood loss, n (%)**			0.954			1.000
<400ml	5703 (99.2)	1669 (99.2)		1540 (99.2)	1540 (99.2)	
≥400ml	47 (0.8)	14 (0.8)		13 (0.8)	13 (0.8)	

In the propensity score matched cohort, the Kaplan–Meier method was used to compare OS and DFS using the log-rank test. The Cox proportional hazards model was used to identify independent prognostic factors in CRC patients. All variables were adjusted using a univariate Cox proportional hazards model. A multivariate Cox proportional hazard model was used in stepwise entry to select prognostic factors. Meanwhile, the hazard ratio and the corresponding 95% confidence interval (CI) were calculated. The densities of infiltrating immune cells between two groups were evaluated using an independent t-test or Mann-Whitney U test. All analyses were performed using IBM SPSS Statistics 25 (SPSS Inc., USA). Statistical significance was set at P < 0.05.

## Results

The results are presented in **Figure 1** and **Table 1**. Our median postoperative follow-up period was 69.4 months (95% CI: 68.7–70.0) for all the patients, 69.5 months (95% CI: 68.8–70.2) for the low preoperative WBC group, and 69.0 months (95% CI: 67.5–70.4) for the high preoperative WBC group (P = 0.677).

Generally, a high WBC classification is defined as a preoperative WBC count of ≥ 7,000/µL. Based on this definition, 22.6% (1,683/7,433) of patients were in the high preoperative WBC group and 77.4% (5,750/7,433) were in the low preoperative WBC group. The patient characteristics are shown in **Table 1**. More men (65.0% *vs*. 57.7%, P < 0.001) were in the high preoperative WBC group than in the low preoperative WBC group. Patients in the high preoperative WBC group were less likely to undergo preoperative adjuvant chemotherapy (5.7% *vs*. 8.9%, P < 0.001) and were more prone to have a tumor located on the left-side of the colon (23.0% *vs*. 20.6%, P < 0.001), right-side colon (27.0% *vs*. 23.8%, P < 0.001), and the transverse colon (can’t tell right or left) (1.6% *vs*. 1.2%, P < 0.001), had more mucinous adenocarcinoma (14.3% *vs*. 11.1%, P < 0.001), signet-ring cell carcinoma (2.0% *vs*. 1.3%, P < 0.001), and positive surgical margins (2.0% *vs*. 1.3%, P < 0.001). Furthermore, the high preoperative WBC group also had had a worse T (P < 0.001) and M stage (P = 0.002), a higher TNM stage (P < 0.001), more infiltrating lymph nodes > 12 (81.6% *vs*. 77.3%, P = 0.001) and more hemoglobin < 90 g/L (8.9% *vs*. 5.8%, P < 0.001). There were no significant differences in age, surgical approach, vascular tumor thrombus, nerve invasion, N stage, or the number of cancer nodules ≥ 1 (P > 0.05). The results indicated that a high preoperative WBC count was more likely to correlate with more malignant clinicopathological features in CRC patients.

**Figure 2:**
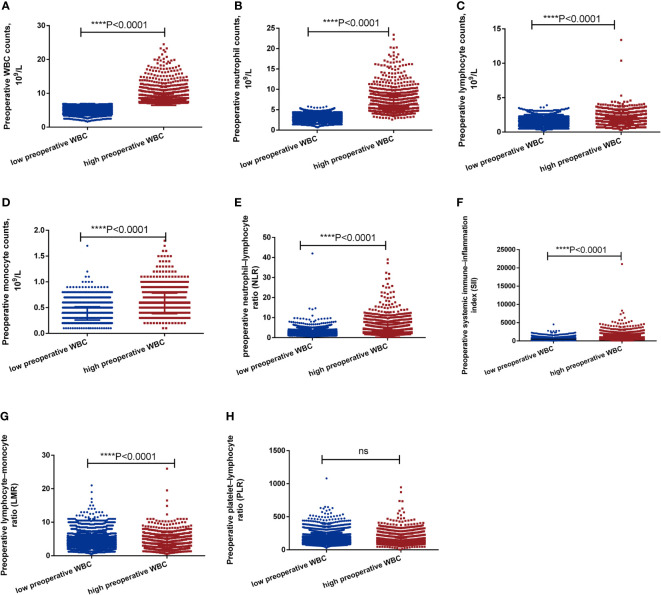
The level of inflammation-related biomarkers between high preoperative WBC group and low preoperative WBC group before matching. **(A-H)**. The level of WBC, neutrophils, lymphocyte, monocyte, NLR, PLR, LMR, SII between high preoperative WBC group and low preoperative WBC group before matching. Differences were considered significant at ****P < 0.0001, compared to the low preoperative WBC group. ns, no significance.

Preoperative WBC counts, neutrophil counts, lymphocyte counts, monocyte counts, neutrophil-lymphocyte ratio (NLR), systemic immune-inflammation index (SII) was markedly higher in the high preoperative WBC group than in the low preoperative WBC group (all P<0.0001, **Figures 2A–F**). However, preoperative lymphocyte-monocyte ratio (LMR) was significantly higher in the low preoperative WBC group than in the high preoperative WBC group (P<0.0001, **Figure 2G**). There were no significant differences in platelet-lymphocyte ratio (PLR) between two groups (**Figure 2H**). It showed that a high preoperative WBC count was associated with more elevated inflammation-related biomarkers.

The propensity score matching was chosen to reduce the imbalance due to the differences in baseline characteristics between the two groups. After matching, 1,553 patients remained in each group. There were no significant differences in patient characteristics between the two groups in the matched cohort, except for surgical margin positivity and preoperative hemoglobin (**Table 1**). In the propensity score matched cohort, the overall mortality rate was significantly higher in the high preoperative WBC count group (26.3% *vs*. 22.2%, P = 0.007) than in the low preoperative WBC count group during follow-up for more than 5 years. Furthermore, a greater percentage of patients in the high preoperative WBC count group required blood transfusion (3.9% *vs*. 1.8%, P = 0.001, **Table 1**). There was no significant difference in blood loss between the two groups (0.8% *vs*. 0.8%, P=1.000, **Table 1**). However, the occurrence of blood transfusion is significantly different, which may be related to the different degree of anemia between the two groups before operation. Summarizing this propensity score matched cohort, a high preoperative WBC count was associated with a higher mortality rate and more blood transfusions after CRC surgery.

**Figure 3:**
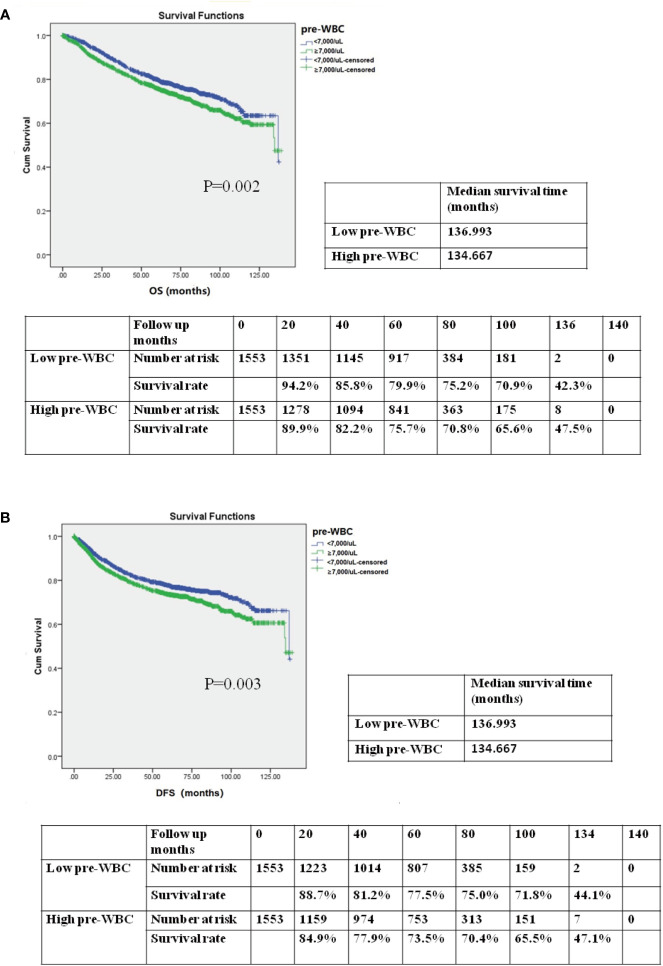
**(A)** Kaplan–Meier survival curve for overall survival (OS) according to preoperative WBC (pre-WBC) in the propensity score-matched cohort. The OS rates, median survival time, and number at risk are shown. **(B)** Kaplan–Meier survival curve for disease-free survival (DFS) according to preoperative WBC (pre-WBC) in the propensity score-matched cohort. The DFS rates, median survival time, and number at risk are shown. Significance with *P* < 0.05.

To assess the association between preoperative WBC count and prognosis, we performed a Kaplan–Meier survival analysis for OS and DFS after propensity score matching. The OS and DFS in the high preoperative WBC group were shorter than those in the low preoperative WBC group (**Figures 3A**, **B**; median survival time in OS: 136.933 months *vs*. 134.667 months; 5-year OS rate: 79.9% *vs*. 75.7%; P = 0.002; median survival time in DFS: 136.933 months *vs*. 134.667 months; 5-year DFS rate: 77.5% *vs*. 73.5%; P = 0.003). After matching, the high preoperative WBC count group exhibited worse outcomes than the low preoperative WBC count group.

In the propensity score matched cohort, the Cox proportional hazards model was created to evaluate the association between the preoperative WBC count and survival, as shown in **Tables 2** and **3**. OS and DFS were compared in a univariate Cox model and later in a multivariate Cox regression. After adjustment, multivariable Cox regression showed that a high preoperative WBC count was strongly associated with poorer OS (hazard ratio, 1.234; 95% CI, 1.068–1.426; P = 0.004) and worse DFS (hazard ratio, 1.210; 95% CI, 1.047–1.397, P = 0.01) when compared to the low preoperative WBC count group. Other adverse prognostic factors for OS and DFS after multivariate analysis were age (≥65 years), preoperative neoadjuvant chemotherapy, tumor histology (signet-ring cell carcinoma), vascular tumor thrombus, nerve invasion, surgical margin positivity, TNM stage (III and IV), infiltrating lymph nodes > 12, number of cancer nodules ≥ 1, and intraoperative blood transfusion. In summary, high preoperative WBC count is an independent predictor of OS and DFS in CRC patients.

**Table 2:** Univariable and multivariable cox regression analysis for overall survival in the propensity score matched cohort.

Variables	UnivariablesHR(95% CI)	P Value	MultivariablesHR((95% CI)	P Value
**Pre-WBC classification**				
<7,000/uL	1		1	
≥7,000/uL	1.247 (1.081-1.440)	0.003	1.234 (1.068-1.426)	0.004
**Sex**				
Male	1			
Female	0.917 (0.787-1.067)	0.262		
**Age**				
≤44	1		1	
45-54	0.989 (0.742-1.318)	0.939	1,197 (0.896-1.601)	0.224
55-64	1.040 (0.797-1.357)	0.773	1.237 (0.945-1.620)	0.122
65-74	1.298 (0.984-1.711)	0.065	1.684 (1.270-2.233)	<0.001
>75	2.222 (1.657-2.979)	<0.001	3.147 (2.331-4.248)	<0.001
**Preoperative Neoadjuvant** **chemotherapy**				
No	1		1	
Yes	1.375 (1.017-1.859)	0.039	1.703 (1.227-2.365)	0.001
**Surgical approach**				
Laparotomy	1			
Laparoscopy	1.003 (0.756-1.330)	0.983		
**Tumor location**				
Rectum	1			
Left-side colon	1.102 (0.919-1.320)	0.294		
Right-side colon	1.068 (0.900-1.267)	0.45		
Entire colon	0.000 (0-8.452E+60)	0.916		
Transverse colon (cant tell right or left)	1.116 (0.554-2.248)	0.76		
**Tumor histology**				
Adenocarcinoma	1		1	
Mucinous adenocarcinoma	0.984 (0.792-1.222)	0.885	1.021 (0.808-1.290)	0.861
Signet-ring cell carcinoma	3.996 (2.656-6.012)	<0.001	1.818 (1.177-2.809)	0.007
**Vascular tumor thrombus**				
Negative	1		1	
Positive	2.598 (2.241-3.012)	<0.001	1.520 (1.281-1.803)	<0.001
**Nerve invasion**				
Negative	1		1	
Positive	2.355 (2.013-2.754)	<0.001	1.601 (1.349-1.900)	<0.001
**TNM stage**				
I	1		1	
II	1.515 (1.115-2.059)	0.008	1.337 (0.980-1.824)	0.067
III	3.190 (2.395-4.248)	<0.001	2.066 (1.522-2.806)	<0.001
IV	12.179 (8.778-16.898)	<0.001	7.408 (5.205-10.545)	<0.001
Unknown	1.009 (0.365-2.788)	0.986	0.926 (0.320-2.683)	0.887
**Surgical margin positivity**				
No	1		1	
Yes	4.591 (3.214-6.558)	<0.001	2.599 (1.798-3.756)	<0.001
**Infiltrating lymph nodes > 12**				
No	1			
Yes	0.758 (0.639-0.900)	0.002	0.828 (0.691-0.994)	0.043
**Number of cancer nodule≥1**				
No	1			
Yes	2.681 (2.276-3.159)	<0.001	1.421 (1.183-1.706)	<0.001
**Blood Transfusion**				
No	1		1	
Yes	1.759 (1.240-2.493)	0.002	1.596 (1.120-2.276)	0.01
**Blood loss**				
<400ml	1			
≥400ml	1.304 (0.619-2.745)	0.485		
**Surgery again within 30 days**				
No	1			
Yes	0.804 (0.464-1.392)	0.435		

**Table 3:** Univariable and multivariable cox regression analysis for disease-free survival in the propensity score matched cohort.

Variables	UnivariablesHR(95% CI)	P Value	Multivariable HR((95% CI)	P Value
**Pre-WBC classification**				
<7,000/uL	1		1	
≥7,000/uL	1.241 (1.075-1.432)	0.003	1.210 (1.047-1.397)	0.01
**Sex**				
Male	1			
Female	0.906 (0.778-1.055)	0.205		
**Age**				
≤44	1		1	
45-54	0.998 (0.749-1.329)	0.987	1.255 (0.939-1.679)	0.125
55-64	1.048 (0.803-1.367)	0.732	1.306 (0.997-1.710)	0.053
65-74	1.273 (0.965-1.678)	0.087	1.696 (1.278-2.249)	<0.001
75+	2.132 (1.590-2.859)	<0.001	2.925 (2.167-3.949)	<0.001
**Preoperative Neoadjuvant chemotherapy**				
No	1		1	
Yes	1.403 (1.038-1.897)	0.028	1.837 (1.322-2.552)	<0.001
**Surgical approach**				
Laparotomy	1			
Laparoscopy	0.965 (0.727-1.279	0.803		
**Tumor location**				
Rectum	1			
Left-side colon	1.105 (0.922-1.324)	0.28		
Right-side colon	1.052 (0.887-1.247)	0.563		
Entire colon	0.000 (0-1.463E+58)	0.912		
Transverse colon (cant tell right or left)	1.069 (0.531-2.154)	0.852		
**Tumor histology**				
Adenocarcinoma	1		1	
Mucinous adenocarcinoma	0.984 (0.792-1.221)	0.881	1.015 (0.803-1.283)	0.9
Signet-ring cell carcinima	3.670 (2.440-5.521)	<0.001	1.820 (1.174-2.821)	0.007
**Vascular tumor thrombus**				
Negative	1		1	
Positive	2.675 (2.307-3.102)	<0.001	1.520 (1.280-1.806)	<0.001
**Nerve invasion**				
Negative	1		1	
Positive	2.405 (2.056-2.813)	<0.001	1.622 (1.365-1.927)	<0.001
**TNM stage**				
I	1		1	
II	1.536 (1.130-2.086)	0.006	1.367 (1.001-1.865)	0.049
III	3.334 (2.503-4.441)	<0.001	2.146 (1.579-2.917)	<0.001
IV	12.929 (9.314-17.947)	<0.001	7.754 (5.442-11.047	<0.001
Unknown	1.005 (0.364-2.778)	0.992	0.938 (0.323-2.719)	0.906
**Surgical Margin positivity**				
No	1		1	
Yes	4.398 (3.079-6.282)	<0.001	2.177 (1.504-3.149)	<0.001
**Infiltrating Lymph nodes > 12**				
No	1		1	
Yes	0.726 (0.612-0.862)	<0.001	0.809 (0.675-0.969)	0.022
**Number of cancer nodules ≥1**				
No	1		1	
Yes	2.754 (2.338-3.245)	<0.001	1.426 (1.187-1.715)	<0.01
**Blood Transfusion**				
No	1		1	
Yes	1.745 (1.231-2.475)	0.002	1.692 (1.186-2.414)	0.004
**Blood loss**				
<400ml	1			
≥400ml	1.398 (0.664-2.942)	0.378		
**Surgery again within 30 days**				
No	1			
Yes	0.818 (0.472-1.417)	0.474		

**Figure 4:**
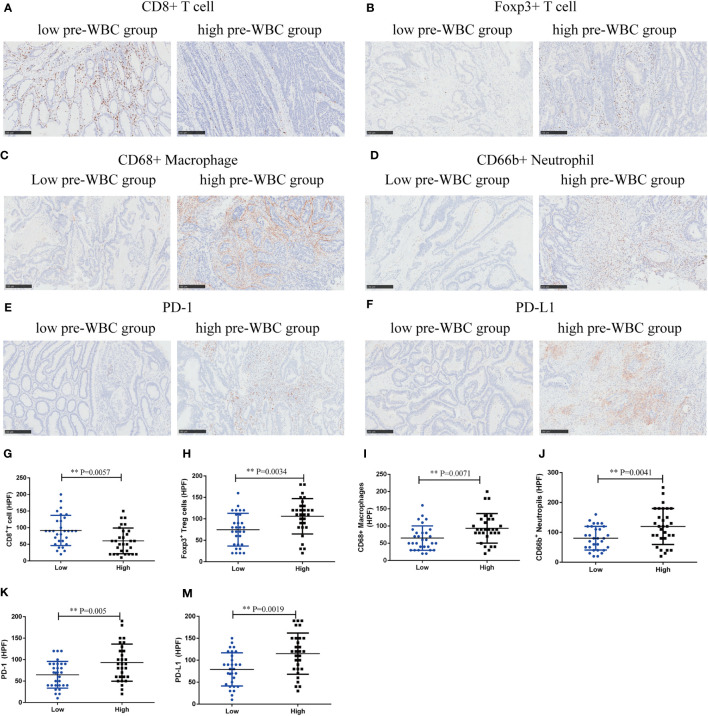
High preoperative WBC group was associated with immunosuppressive contexture in CRC. **(A–F)**. Representative immunohistochemical (IHC) staining of CD8^+^ T cells, Foxp3^+^ Tregs, CD68^+^ Macrophages, CD66b^+^ Neutrophils, and immunosuppressive checkpoints (PD-1, PD-L1) between high and low preoperative WBC groups. **(G–M)** Comparison of CD8^+^ T cells, Foxp3^+^ Tregs, CD68^+^ Macrophages, CD66b^+^ neutrophils and immunosuppressive checkpoints (PD-1, PD-L1) between two groups. n=30 in each group. Differences were considered significant at **P < 0.01, compared to the low preoperative WBC group.

To explore the potential mechanism, we performed immunohistochemical staining of tumor-infiltrating immune cells in the high and low preoperative WBC groups (n = 60). Compared with the low preoperative WBC group, the high preoperative WBC group showed higher expression of Treg cells (P = 0.0034), CD68^+^ macrophages (P = 0.0071), and CD66b^+^ neutrophils (P = 0.0041), but lower expression of CD8^+^ T cells (P = 0.0057), suggesting a more immunosuppressive TME with increased Treg cells, macrophages, and neutrophil infiltration in the high preoperative WBC group (**Figures 4A–D, G–J**). We also found that the expression of PD-1 and PD-L1 increased in the high preoperative WBC count group (P = 0.005; P = 0.0019) (**Figures 4E–F, K–M**). Taken together, these data suggest that a high preoperative WBC count is associated with an immunosuppressive environment in CRC.

## Discussion

Even after the use of propensity score matching, our findings suggested that the high preoperative WBC count group exhibited worse outcomes than the low preoperative WBC count group. We confirmed that high preoperative WBC count is an independent prognostic indicator for predicting OS and DFS in CRC patients. Additionally, the analysis showed that age (≥65 years), preoperative neoadjuvant chemotherapy, tumor type (signet-ring cell carcinoma), vascular tumor thrombus, nerve invasion, positive surgical margin, stage of TNM (III and IV), infiltrating lymph nodes > 12, number of cancer nodules ≥ 1, and intraoperative blood transfusion were also independent risk factors for worse survival outcomes. A high preoperative WBC count was associated with an immunosuppressive environment in CRC, with higher infiltration of Treg cells, neutrophils, and macrophages and increased levels of PD-1 and PD-L1, but less infiltration of CD8^+^ T cells. Overall, this study demonstrated that a high WBC count was independently linked with worse outcomes and an immunosuppressive environment in CRC.

Research has shown that up to 50% of cancers may be associated with inflammation, which is involved in the initiation, promotion, malignant progression, invasion, and metastasis of cancer (22). Our study indicated that leukocytosis before surgery was present in 22.6% of CRC patients and was strongly associated with worse OS and DFS. Several studies have shown that multiple types of malignant tumors are related to inflammation or infection, such as lung, gastric, and skin cancer (23–25). In addition, the host inflammation reaction can inhibit antitumor immune function, thereby leading to poor prognosis of patients (26). At present, the correlation between higher levels of circulating inflammatory markers and prognosis has been revealed in various malignant tumors (27–30). Inflammation is an immune response characterized by a dramatic increase in the number of leukocytes in circulation and infectious tissue (31). Various studies concluded that inflammatory markers such as the neutrophil/lymphocyte ratio, platelet/lymphocyte ratio, and C-reactive protein can all predict the prognosis of CRC (32, 33). Although several groups have reported adverse effects of peripheral leukocytosis on the prognosis of patients with various malignant tumors, this is limited by a retrospective mismatch, uneven treatment options, short follow-up, lack of multivariable analysis, and a small number of patients (5, 15, 19, 34). Our larger sample-matched cohort validated that a higher preoperative WBC count was correlated with poorer OS and DFS in CRC, which was in line with previous research (7, 35). Multivariate analysis validated that a high preoperative WBC count was an independent prognostic marker for malignancy in CRC. Our results showed that the mortality rate of patients with leukocytosis increased by 4.1%. Moreover, preoperative leukocytosis is associated with increased mortality, morbidity, and postoperative complications in CRC surgery (36, 37). Therefore, based on the potential role of leukocytosis in predicting prognosis, it could be used for disease management and follow-up to improve the OS of CRC patients.

Immunomodulatory cytokines and systemic inflammatory markers play a key role in the occurrence and development of cancer. The mechanism underlying the relationship between systemic inflammation and survival outcomes in CRC patients remains unclear. The TME may be one of the main factors involved in its pathogenesis. Many inflammatory cells represent innate and acquired immune responses in the microenvironment of solid malignant tumors (38). Our study used CD8 as a cytotoxic T cell marker, Foxp3 as a Treg cell marker, CD66b as a neutrophil marker, and CD68 as a macrophage marker, which are the key components of the TME. Our study showed that a high preoperative WBC count was associated with an immunosuppressive environment in CRC, with higher infiltration of Treg cells, neutrophils, and macrophages and increased levels of PD-1 and PD-L1, but less infiltration of CD8^+^ T cells. Several studies have reported that a high baseline WBC count is associated with a low infiltration of CD8^+^ T cells in tumors, which is consistent with the findings of our study (5, 7). However, to the best of our knowledge, there are no reports on the functional relevance of leukocytosis in the immunosuppressive TME in CRC; thus, our research fills this gap. Simultaneously, we provided a reasonable explanation for the relationship between leukocytosis and poor prognosis.

Several studies have shown that the immune microenvironment in tumors is closely related to clinical outcomes and therapeutic drug resistance (39). Treg cells play a role in the promotion of tumors by inhibiting adaptive antitumor immunity (40). Tumor growth factors, pro-inflammatory cytokines, pro-angiogenic factors, and reactive oxygen species rich in the TME, together with a large number of Treg cells, can lead to cytotoxic T-lymphocyte dysfunction and poor prognosis (41). Tumor-associated macrophages, which tend to be pro-tumor M2 subsets, may induce cancer cell proliferation by secreting growth factors and angiogenesis. They may also promote tumor growth by secreting matrix metalloproteinases (42, 43). Neutrophils are crucial regulators of both inflammation and immune responses, accounting for 50–70% of leukocytes in the circulation, and are the major elements of WBC (44). Neutrophils can be transformed into a tumor-promoting state in the TME (45). In addition to cytotoxicity, neutrophils can promote the spread of tumor cells by secreting metalloproteinases and elastase to degrade the extracellular matrix. It also regulates immunosuppression by secreting reactive oxygen species and arginase-1, thus limiting T cell-dependent antitumor immunity (46). It is an immunosuppressive TME that leads to poor prognosis in CRC.

Clinically, the TNM staging system is the most used indicator for risk stratification of CRC patients and guides treatment decisions (47). However, TNM staging is often performed postoperatively, and it is difficult to predict survival preoperatively and choose further treatment strategies. Moreover, TNM staging can only reflect the biological behavior of tumors. However, patients tend to have different survival outcomes even at the same TNM stage (48). The prognosis of tumors is not only related to the clinicopathological characteristics of the tumor but is also affected by tumor-host interactions, including inflammatory and immune responses (49). Recently, owing to the repeatable, inexpensive, and convenient features of hematological indices, inflammatory markers established on the basis of blood cell counts have been regarded as potential prognostic indicators for CRC patients (50). These inflammatory indicators are helpful for anesthesiologists and surgeons for comprehensive evaluation before surgery. The first contribution of our study is that we can predict the prognosis of CRC patients using a simple and feasible method before surgery. At present, anesthesiologists and surgeons are paying increasing attention to the short- and long-term prognosis of patients (51–53). Enhanced recovery after surgery also requires anesthesiologists to focus on these factors (54). This suggests that anesthesiologists and surgeons should pay more attention to patients with a higher preoperative inflammation status and take measures to inhibit perioperative stress response and inflammation to improve the prognosis of patients (55–57). This implies good clinical application of these implications. The second contribution of our research is that we are the first to report that leukocytosis is associated with an immunosuppressive microenvironment in CRC, which will provide a better understanding of the relationship between leukocytosis and worse outcomes and help us tailor more precise strategies for CRC patients.

Thus, our research has important implications. The advantage of our clinical research is that our overall sample size (>7000) and allocation (>1500) in each group is much larger than those in previous studies (5, 10, 15, 19, 20), with data from one of the largest cancer centers in China. Another advantage of this study is that we focused on the long-term prognosis of CRC patients. Our median postoperative follow-up period was >5 years (median: 69.6 months), which is much longer than that in previous studies (5, 15, 19). Additionally, we used propensity score matching and multiple Cox regression analyses to correct for confounding factors. Therefore, our study provides high-quality evidence. Previous studies have suggested that leukocytosis is associated with decreased levels of CD8^+^ T cells in the CRC TME; however, the evaluation of other immunosuppressive cells has not been reported. We found that the preoperative WBC count was correlated with several immunosuppressive cells, shaping an immunosuppressive TME. Our study not only explored a new mechanism behind the clinical significance of preoperative WBC count in CRC malignancy but also stratified patients for personalized treatment. Further research is needed to determine whether leukocytosis could be an immunological biomarker in patients who are sensitive to immunotherapy.

Our study had some limitations. First, the study was retrospective and non-randomized, and patient information was obtained from a cancer center. Second, due to the various perioperative factors associated with leukocytosis, we were unable to eliminate the potential effects of unmeasured confounding factors. Third, although we have determined the effect of leukocytosis on the tumor immunosuppressive microenvironment in the malignant process of CRC, the potential mechanism of these immune cell interactions needs to be further studied. Well-designed prospective and basic studies are helpful to explore the clinical significance and immune environment of preoperative leukocytosis in the long-term prognosis of CRC patients.

In conclusion, preoperative leukocytosis was independently associated with increased overall mortality and cancer recurrence after CRC surgery, and it was associated with an immunosuppressive TME, which might be useful for risk stratification and follow-up scheduling.

## Data availability statement

The original contributions presented in the study are included in the article/**supplementary material**. Further inquiries can be directed to the corresponding authors.

## Ethics statement

This retrospective study was approved by the ethics committee of the Shanghai Cancer Center at Fudan University in Shanghai, China (IRB2105235-6). The patients/participants provided their written informed consent to participate in this study.

## Author contributions

MW, MS, DZ, and CM designed the study; MW, YY, MG, and WZ performed the study; KN and QL analyzed the data; and MW, WZ, and MG wrote the paper. MW and CM revised the paper. All authors contributed to the article and approved the submitted version.

## Funding

Our study was supported by the National Natural Science Foundation of China (No.82002538, 82072213); Shanghai Pujiang Talent Plan (No. 2020PJD013); Clinical Research Plan of SHDC (No. SHDC2020CR1005A); National Key Research and Development Program of China (No. 2020YFC2008400) and Wu Jieping Medical Foundation Clinical Research Special funding.

## Acknowledgments

We thank Changming Zhou (Department of Cancer Prevention, Shanghai Cancer Center, Fudan University) for providing statistical analysis and consultation.

## Conflict of interest

The authors declare that the research was conducted in the absence of any commercial or financial relationships that could be constructed as a potential conflict of interest.

## Publisher’s note

All claims expressed in this article are solely those of the authors and do not necessarily represent those of their affiliated organizations, or those of the publisher, the editors and the reviewers. Any product that may be evaluated in this article, or claim that may be made by its manufacturer, is not guaranteed or endorsed by the publisher.
