# Supplementary material for: High preoperative white blood cell count determines poor prognosis and is associated with an immunosuppressive microenvironment in colorectal cancer
Source: Front Oncol. 2022 Jul 29;12:943423. doi: 10.3389/fonc.2022.943423 (PMC9373020; doi:10.3389/fonc.2022.943423)
Supplement: Supplementary file 1 [file Table_1.docx]

**Supplementary Table 1. Immunohistochemistry antibodies**

| **Antibody name** | **Description** | **Manufacturer** | **Catalog No.** | **Dilution** | **Identical cells** |
| --- | --- | --- | --- | --- | --- |
| Anti-CD8 alpha antibody | Rabbit monoclonal | Abcam | Ab237709 | 1:200 | CD8^+^ T cells |
| Anti-Foxp3 antibody | Mouse monoclonal | Abcam | Ab450 | 1:50 | Tregs (regulatory T cells) |
| Anti-CD68 antibody | Rabbit  monoclonal | Abcam | Ab283654 | 1:100 | CD68^+^ Macrophage cells |
| Anti-CD66b antibody | Rabbit  polyclonal | Abcam | Ab214175 | 1:250 | CD66b^+^ Neutrophil cells |
| Anti-PD-1 antibody | Rabbit monoclonal | Abcam | Ab137132 | 1:250 | PD-1^+^ cells |
| Anti-PD-L1 antibody | Rabbit monoclonal | Abcam | Ab213524 | 1:250 | PD-L1^+^ cells |
